# Study on the identification of *Pinelliae rhizoma* and *Pinelliae pedatisectae rhizoma* based on the characteristic component triglochinic acid

**DOI:** 10.1039/c9ra01626k

**Published:** 2019-04-16

**Authors:** Yong Jing, Yueyue Lai, Hui Chen, Min Li, Juan Zhou, Zelun Lan

**Affiliations:** Key Laboratory of Standardization of Chinese Herbal Medicine, State Key Laboratory Breeding Base of Systematic Research, Development and Utilization of Chinese Medicine Resources, Ministry of Education, College of Pharmacy, Chengdu University of Traditional Chinese Medicine Chengdu 611137 China limin@cdutcm.edu.cn +86-13980038316; Sichuan Institute for Food and Drug Control Chengdu 611731 China; Sichuan Neautus Traditional Chinese Medicine Co., Ltd. Chengdu 611731 China

## Abstract

For the first time, a monomeric compound, triglochinic acid, has been isolated from the tubers of *Pinellia pedatisecta* Schott with its structure analyzed using NMR. Its molecular formula is C_7_H_8_O_6_, with the chemical name (2*E*)-but-2-ene-1,2,4-tricarboxylic acid. Through HPLC-DAD and HPLC-MS analysis studies, it has concluded that *Pinelliae rhizoma* does not contain triglochinic acid, while *Pinelliae pedatisectae rhizoma* does. This key observation was used as a characteristic component to distinguish these two herbs. We analyzed 39 batches of *Pinelliae rhizoma* collected in herbal medicine market, among which triglochinic acid was detected in 27 batches, resulting in a adulteration ratio of *Pinelliae rhizoma* reaching 69.2%. Our method demonstrates great potential for authenticating the products, thus ensuring the quality of *Pinelliae rhizoma*.

## Abstract 2

A monomeric compound, triglochinic acid, has been isolated from *Pinelliae pedatisectae rhizoma* with its structure analyzed using NMR. It is used as a characteristic compound to distinguish *Pinelliae pedatisectae rhizoma* and *Pinelliae rhizoma* for quality control.

## Graphical abstract

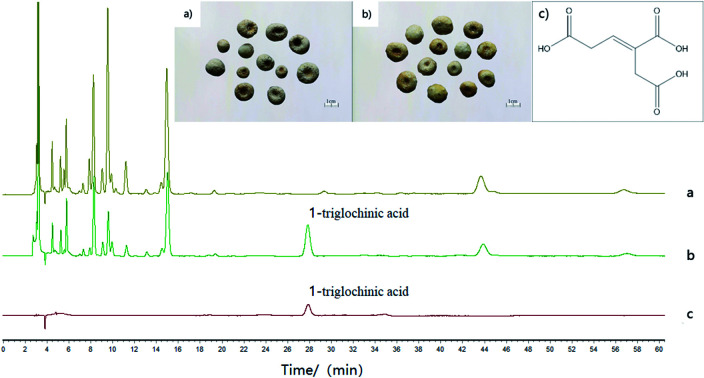

## 1. Introduction

*Pinelliae rhizoma* is the dry tubers of *Pinellia ternata* (Thunb.) Breit.^1^ It was documented as Diwen or Shuiyu in Shennong's Materia Medica (Shen Nong Ben Cao Jing), and other medical classics afterwards. During ancient times, counterfeit or fake products of *Pinelliae rhizoma* existed, such as *Arisaema ringens* (Thunb.) Schott in Tang Ben Cao by Su Gong and *Pinellia pedatisecta* Schott in Tu Jing Ben Cao. In modern times, *Pinelliae rhizoma* in rural areas was replaced by herbs of the same genus like *Typhonii flagelliformis* and *Arisaema heterophyllum* Blume according to Modern Chinese Materia Medica.^2^ Recently, *Pinelliae rhizoma* was discovered to be mixed with a large amount of *Pinelliae pedatisectae rhizoma* in the market, which greatly affected the quality of the herbs. *Pinelliae pedatisectae rhizoma*, also known as the palm leaf Pinellia and South Star, is the dry tubers of *Pinellia pedatisecta* Schott, documented in the Chinese Materia Medica Standards of Shandong, Hubei and Jiangsu,^3–5^ as well as in the Dictionary of Traditional Chinese Medicine^6^ and Chinese Flora.^7^

*Pinelliae rhizoma* and *Pinelliae pedatisectae rhizoma* are both of the *Pinellia* genus, which can easily be identified by the leaves and flowers.^7^ However, their medicinal materials are extremely similar. *Pinelliae rhizoma* is spheroidal in shape, while *Pinelliae pedatisectae rhizoma* is also spheroidal with several small bulbs alongside. If the small bulbs of *Pinelliae pedatisectae rhizoma* are not yet formed or removed during processing, the herbs will be very similar to *Pinelliae rhizoma*, which makes them difficult to distinguish. *Pinelliae rhizoma* enjoys a wide range of clinical applications, being the raw material in 489 patent traditional Chinese medicine^8^ and in 3029 prescriptions.^9^ The growing period of *Pinellia ternata* is 4–5 months with high cost and low yield, and its market price is 85–120 yuan per kilogram. On the other hand, the clinical application of *Pinelliae pedatisectae rhizoma* is limited, only used in a few prescriptions. Its lifespan is shorter, about 3–4 months with lower cost, higher yield and the price is 40–50 yuan per kilogram. Therefore, the interests drive people to incorporate *Pinelliae pedatisectae rhizoma* into *Pinelliae rhizoma*.

Nowadays, the identifications of *Pinelliae rhizoma*, *Typhonii flagelliformis rhizoma* and *Arisaematis rhizoma* are mostly based on morphology identification, thin layer chromatography identification, and fingerprint identification.^10–12^ However, the identification studies on *Pinelliae rhizoma* and *Pinelliae pedatisectae rhizoma* are very limited. In particular, it is hard to identify the incorporation of *Pinelliae pedatisectae rhizoma* in *Pinelliae rhizoma*. From 2016 to 2017, we collected *Pinelliae rhizoma* from several herbal medicine markets: Hehuachi, Anguo, Bozhou, Qingping, Yulin, *etc.* However, more than 60% of the samples were counterfeit. This situation urges us to establish an effective and rapid analytical method that can detect fake herbs and improve the quality control of *Pinelliae rhizoma*, meanwhile laying the foundation of a standard herbal medicine market.

## 2. Material

### 2.1 Equipment

Agilent 1200 high performance liquid chromatography (with DAD detector), Agilent 1290/6460 LC/MS, Agilent ZORBAX Eclipse Plus C_18_ column (4.6 × 250 mm, 5 μm), Agilent ZORBAX Eclipse Plus C_18_ column (2.1 × 50 mm, 1.8 μm), XB-C_18_ column (80 × 250 mm, 10 μm), 3–30 K centrifuge (Sigma, Germany), KQ-500DE CNC ultrasonic cleaner (Kunshan Ultrasonic Instrument Co., Ltd.); ULUP-IV-10T Youpu series ultrapure water (Chengdu Ultrapure Technology Co., Ltd.), SQP electronic balance (Sartorious Scientific Instrument Beijing Co., Ltd.).

### 2.2 Reagents

Triglochinic acid reference substance (96.68% purity, batch number 20170201), purchased from Chengdu Push Bio-Technology Co., Ltd. Phosphoric acid (Chengdu Kelon Chemical Reagent Factory), formic acid (Tianjin Kemiou Chemical Reagent Co., Ltd.) and acetonitrile (Thermo Fisher Scientific (China)) are chromatographically pure, and the water is ultrapure.

### 2.3 Medicinal materials

**Fig. 1:**
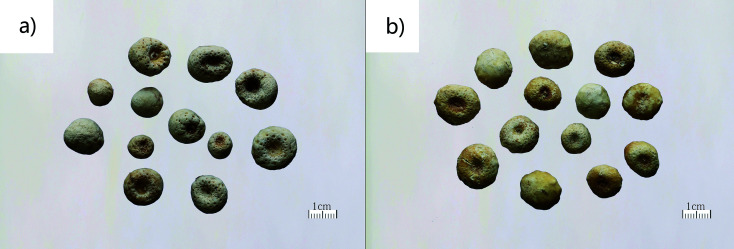
Photos of *Pinelliae rhizoma* (a) and *Pinelliae pedatisectae rhizoma* (b).

From Sichuan, Gansu, Guizhou, Hebei, Shanxi, Chongqing and other provinces and cities, we collected 28 batches of *Pinelliae rhizoma*. Also from Hebei, Heilongjiang, Gansu, Sichuan and other places, we collected 16 batches of *Pinelliae pedatisectae rhizoma*. All the materials were determined by Professor Min Li from College of Pharmacy, Chengdu University of Traditional Chinese Medicine. Information of these samples is shown in Table 1. Photos of *Pinelliae rhizoma* and *Pinelliae pedatisectae* are shown in Fig. 1.

**Table 1:** Market Information of the samples

ID	Sample	Origin
BX1	*Pinelliae rhizoma*	Muba, Nanchong, Sichuan
BX2	*Pinelliae rhizoma*	Muba, Nanchong, Sichuan
BX3	*Pinelliae rhizoma*	Jinchuan, Aba, Sihuan
BX4	*Pinelliae rhizoma*	Pengxi, Suining, Sichuan
BX5	*Pinelliae rhizoma*	Wenjiang, Chengdu, Sichuan
BX6	*Pinelliae rhizoma*	Nanjiang, Bazhong, Sichuan
BX7	*Pinelliae rhizoma*	Mianning, Liangshan, Sichuan
BX8	*Pinelliae rhizoma*	Mianning, Liangshan, Sichuan
BX9	*Pinelliae rhizoma*	Hongjiang, Suining, Sichuan
BX10	*Pinelliae rhizoma*	Changle, Nanchong, Sichuan
BX11	*Pinelliae rhizoma*	Shayang, Jinmen, Hubei
BX12	*Pinelliae rhizoma*	Xihe, Longnan, Gansu
BX13	*Pinelliae rhizoma*	Li, Longnan, Gansu
BX14	*Pinelliae rhizoma*	Qingshui, Tianshui, Gansu
BX15	*Pinelliae rhizoma*	Xihe, Longnan, Gansu
BX16	*Pinelliae rhizoma*	Qingshui, Tianshui, Gansu
BX17	*Pinelliae rhizoma*	Weining, Guizhou
BX18	*Pinelliae rhizoma*	Dafang, Guizhou
BX19	*Pinelliae rhizoma*	Hechuan, Chongqing
BX20	*Pinelliae rhizoma*	Shenze, Shijiazhuang, Hebei
BX21	*Pinelliae rhizoma*	Shenze, Shijiazhuang, Hebei
BX22	*Pinelliae rhizoma*	Shenze, Shijiazhuang, Hebei
BX23	*Pinelliae rhizoma*	Houma, Shanxi
BX24	*Pinelliae rhizoma*	Houma, Shanxi
BX25	*Pinelliae rhizoma*	Houma, Shanxi
BX26	*Pinelliae rhizoma*	Weining, Guizhou
BX27	*Pinelliae rhizoma*	Hezhang, Guizhou
BX28	*Pinelliae rhizoma*	Hezhang, Guizhou
HZ1	*Pinelliae pedatisectae rhizoma*	Qizhou, Anguo, Hebei
HZ2	*Pinelliae pedatisectae rhizoma*	Zhengzhang, Anguo, Hebei
HZ3	*Pinelliae pedatisectae rhizoma*	Zhengzhang, Anguo, Hebei
HZ4	*Pinelliae pedatisectae rhizoma*	Xifuluo, Anguo, Hebei
HZ5	*Pinelliae pedatisectae rhizoma*	Qizhou, Anguo, Hebei
HZ6	*Pinelliae pedatisectae rhizoma*	Qizhou, Anguo, Hebei
HZ7	*Pinelliae pedatisectae rhizoma*	Tongnan, Nehe, Heilongjia
HZ8	*Pinelliae pedatisectae rhizoma*	Anguo, Hebei
HZ9	*Pinelliae pedatisectae rhizoma*	Anguo, Hebei
HZ10	*Pinelliae pedatisectae rhizoma*	Anguo, Hebei
HZ11	*Pinelliae pedatisectae rhizoma*	Tongnan, Nehe, Heilongjia
HZ12	*Pinelliae pedatisectae rhizoma*	Anguo, Hebei
HZ13	*Pinelliae pedatisectae rhizoma*	Muba, Nanchong, Sichuan
HZ14	*Pinelliae pedatisectae rhizoma*	Xihe, Longnan, Gansu
HZ15	*Pinelliae pedatisectae rhizoma*	Wenjiang, Chengdu, Sichuan
HZ16	*Pinelliae pedatisectae rhizoma*	Li, Longnan, Gansu

In addition, 39 batches of commercial *Pinelliae rhizoma* were collected from the herbal medicine markets of Hehuachi Chengdu, Anguo Hebei, Yinzhou Anhui, Qingping Guangdong, and Yulin Guangxi. Information of the commercial *Pinelliae rhizoma* samples is shown in Table 2.

**Table 2:** Market information of the commercial herb samples

ID	Sample	Market
SSBX1	Commercial *Pinelliae rhizoma*	Bozhou, Anhui
SSBX2	Commercial *Pinelliae rhizoma*	Bozhou, Anhui
SSBX3	Commercial *Pinelliae rhizoma*	Yulin, Guanxi
SSBX4	Commercial *Pinelliae rhizoma*	Hehuachi, Chegndu
SSBX5	Commercial *Pinelliae rhizoma*	Hehuachi, Chegndu
SSBX6	Commercial *Pinelliae rhizoma*	Hehuachi, Chegndu
SSBX7	Commercial *Pinelliae rhizoma*	Hehuachi, Chegndu
SSBX8	Commercial *Pinelliae rhizoma*	Hehuachi, Chegndu
SSBX9	Commercial *Pinelliae rhizoma*	Hehuachi, Chegndu
SSBX10	Commercial *Pinelliae rhizoma*	Hehuachi, Chegndu
SSBX11	Commercial *Pinelliae rhizoma*	Hehuachi, Chegndu
SSBX12	Commercial *Pinelliae rhizoma*	Hehuachi, Chegndu
SSBX13	Commercial *Pinelliae rhizoma*	Hehuachi, Chegndu
SSBX14	Commercial *Pinelliae rhizoma*	Hehuachi, Chegndu
SSBX15	Commercial *Pinelliae rhizoma*	Anguo, Hebei
SSBX16	Commercial *Pinelliae rhizoma*	Anguo, Hebei
SSBX17	Commercial *Pinelliae rhizoma*	Anguo, Hebei
SSBX18	Commercial *Pinelliae rhizoma*	Anguo, Hebei
SSBX19	Commercial *Pinelliae rhizoma*	Anguo, Hebei
SSBX20	Commercial *Pinelliae rhizoma*	Anguo, Hebei
SSBX21	Commercial *Pinelliae rhizoma*	Anguo, Hebei
SSBX22	Commercial *Pinelliae rhizoma*	Bozhou, Anhui
SSBX23	Commercial *Pinelliae rhizoma*	Bozhou, Anhui
SSBX24	Commercial *Pinelliae rhizoma*	Bozhou, Anhui
SSBX25	Commercial *Pinelliae rhizoma*	Bozhou, Anhui
SSBX26	Commercial *Pinelliae rhizoma*	Bozhou, Anhui
SSBX27	Commercial *Pinelliae rhizoma*	Bozhou, Anhui
SSBX28	Commercial *Pinelliae rhizoma*	Zhejiang
SSBX29	Commercial *Pinelliae rhizoma*	Qingping, Guangdong
SSBX30	Commercial *Pinelliae rhizoma*	Qingping, Guangdong
SSBX31	Commercial *Pinelliae rhizoma*	Qingping, Guangdong
SSBX32	Commercial *Pinelliae rhizoma*	Qingping, Guangdong
SSBX33	Commercial *Pinelliae rhizoma*	Qingping, Guangdong
SSBX34	Commercial *Pinelliae rhizoma*	Qingping, Guangdong
SSBX35	Commercial *Pinelliae rhizoma*	Anguo, Hebei
SSBX36	Commercial *Pinelliae rhizoma*	Anguo, Hebei
SSBX37	Commercial *Pinelliae rhizoma*	Anguo, Hebei
SSBX38	Commercial *Pinelliae rhizoma*	Anguo, Hebei
SSBX39	Commercial *Pinelliae rhizoma*	Anguo, Hebei

## 3. Methods and results

After a systematic study on the chemical components of *Pinelliae rhizoma* and *Pinelliae pedatisectae rhizoma*, we discovered a characteristic peak of *Pinelliae pedatisectae rhizoma* in the HPLC chromatogram of organic acid part, distinguishable from that of *Pinelliae rhizoma*.

### 3.1 Extraction, separation and purification of the characteristic component

Take the powder of *Pinelliae pedatisectae rhizoma* (after passing the no. 4 sieve), and add 10 times the amount of water. Sonicate the sample for 3 times, 1 hour each time. Then, combine the extracts and add phosphoric acid, 1% of the extract volume. Shake and add an equal volume of ethyl acetate for extracting 3 times. Recover the ethyl acetate to obtain the concentrated liquid before dilute it with water. Pass through the XB-C_18_ (80 × 250 mm, 10 μm) preparative column at flow rate of 140 mL per minute, using 5% acetonitrile (containing 0.2% phosphoric acid solution) as the mobile phase, collect the eluent from 19.5 to 22.6 minutes. Then, recover the solvent and change the mobile phase to 5% acetonitrile (containing 0.1% formic acid solution). Use the column again and collect the eluate from 17.4 to 20.3 minutes. Finally, lyophilize the eluate under reduced pressure to obtain the white compound in solid form.

### 3.2 Structural identification of the characteristic component

We used NMR to analyze the structure of the white compound. The data was listed in Table 3. ^1^H-NMR (600 MHz, MeOD-*d*_4_) showed that the compound has five hydrogen signals: a double bond hydrogen signal *δ*_H_ 7.13 (1H, t, *J* = 7.2 Hz) in the low field, two sets of hydrogen signals *δ*_H_ 3.35 (2H, s), 3.27 (2H, d, *J* = 7.2 Hz) in the high field. Correspondingly, 13C-NMR showed 7 carbon signals: 3 carbonyl carbon signals *δ*_C_ 172.9, 172.3, 168.5, a double bond signal *δ*_C_ 136.9, 128.4 in the low field, *δ*_C_ 33.3, 31.7 in the high field. Combined with DEPT 135°, *δ*_C_ 136.9 is a methine, suggesting the double bond is trisubstituted, while *δ*_C_ 33.3, 31.7 is methylene. HSQC carries out hydrocarbon partial assignment: *δ*_C_ 136.9 (*δ*_H_ 7.13), *δ*_C_ 33.3 (*δ*_H_ 3.27), *δ*_C_ 31.7 (*δ*_H_ 3.35), and the link order of carbon is determined by HMBC correlation: *δ*_H_ 7.13 (*δ*_C_ 136.9, C-3) is related to *δ*_C_ 172.3 (C-1), 168.5 (C-5), 128.4 (C-4), and 31.7 (C-1′). *δ*_H_ 3.27 (*δ*_C_ 33.3, C-2) is related to *δ*_C_ 172.3 (C-1), 136.9 (C-3), and 128.4 (C-4). *δ*_H_ 3.35 (*δ*_C_ 31.7, C-1′) is related to C 172.9 (C-2 ′), 168.5 (C-5), 136.9 (C-3), and 128.4 (C-4).

**Table 3:** Carbon and hydrogen assignments of the compound (MeOD-*d*_4_, *δ* ppm, *J* = Hz)

No.	*δ*_C_ (150 MHz)	*δ*_H_ (600 MHz)
1	172.3	
2	33.3	3.27 d, *J* = 7.2
3	136.9	7.13 t, *J* = 7.2
4	128.4	
5	168.5	
1′	31.7	3.35 s
2′	172.9	

**Fig. 2:**
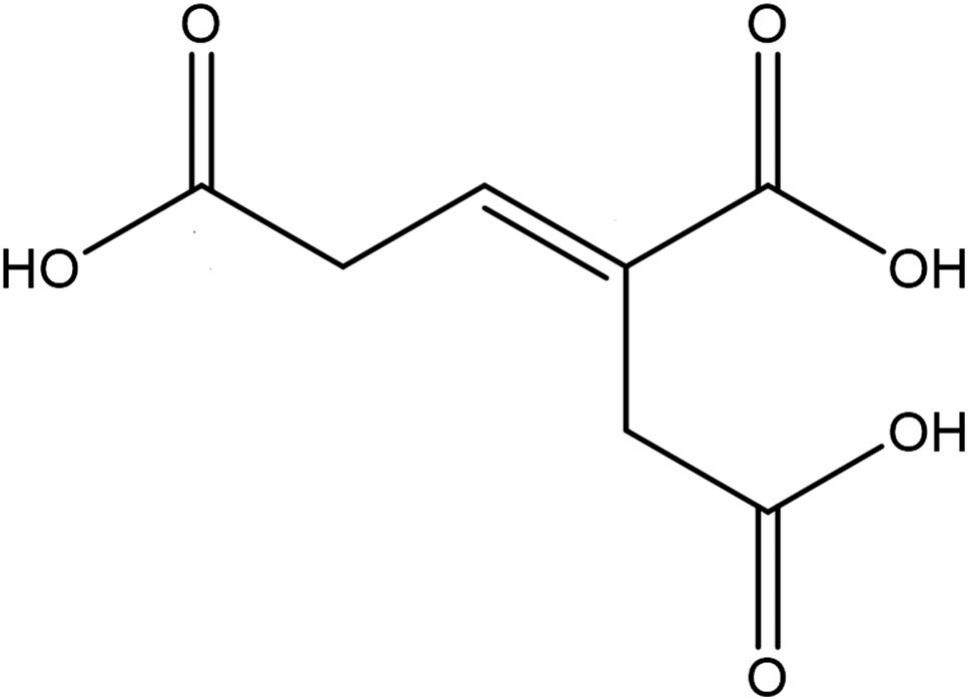
Structural formula of triglochinic acid.

The nuclear magnetic data of this compound is generally consistent with that of triglochinic acid as reported in the literature.^13^ Therefore, this compound was identified as triglochinic acid, a white powdery solid, soluble in water and methanol, with the molecular formula C_7_H_8_O_6_. The chemical name is (2*E*)-but-2-ene-1,2,4-tricarboxylic acid, CAS number: 31795-12-7. Its relative molecular mass is 188.13, boiling point (557.3 ± 50) °C. The structure of triglochinic acid is depicted in Fig. 2.

### 3.3 Selection of the detection wavelength

**Fig. 3:**
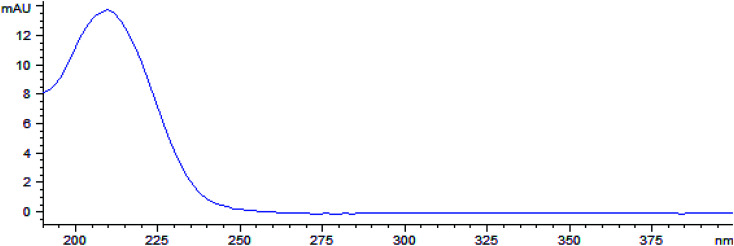
Ultraviolet spectrum of triglochinic acid.

With full-wavelength ultraviolet scanning, the maximum absorption wavelength of triglochinic acid is 210 nm. Therefore, 210 nm was selected as the detection wavelength. The spectrum is shown in Fig. 3.

### 3.4 Chromatographic conditions

**Fig. 4:**
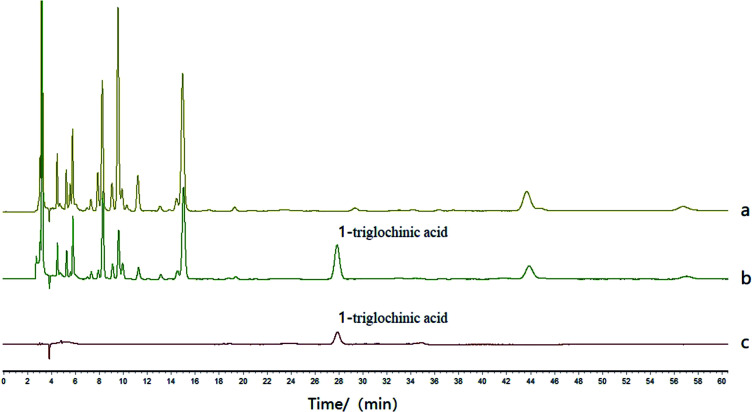
Chromatogram of *Pinelliae rhizoma* (a), *Pinelliae pedatisectae rhizoma* (b) and triglochinic acid, the reference compound (c). The marked peak 1 is triglochinic acid.

The column was an Agilent ZORBAX Eclipse Plus C_18_ column (4.6 × 250 mm, 5 μm). Acetonitrile (A) and 0.1% phosphoric acid solution (B) were applied as the mobile phase, using gradient elution (0–40 min, 1% A; 40–45 min, 1–10% A; 45–50 min, 10–1% A; 50–60 min, 1% A; 30–35 min, 10% A; 35–40 min, 10–0% A; 40–50 min, 0% A) at the flow rate of 0.8 mL per minute, 25 °C column temperature, and 210 nm detection wavelength. The injection volume was 10 μL. The result is shown in Fig. 4.

### 3.5 Solution preparation

Preparation of the reference solution: accurately weigh an appropriate amount of triglochinic acid. Add water to make the reference solution with a concentration of 0.25 μg mL^−1^.

Preparation of the test solution: accurately weigh about 1 g of the sample powder (after passing through the no. 4 sieve) and place it in an Erlenmeyer flask with 20 mL of water. Weigh before and after 45 minutes of sonication (250 W, 40 kHz), add the lost weight with water. Filter the solution and transfer 10 mL of the filtrate to a 50 mL centrifuge tube. Add 0.1 mL phosphoric acid and shake, then add 20 mL ethyl acetate and shake. Centrifuge (5000 rpm) the solution, and aspirate the upper liquid ethyl acetate. Extract acid solution with ethyl acetate for three more times, 20 mL each time. Then combine the ethyl acetate solution and evaporate the solvent to dry under reduced pressure. Add 2 mL acetonitrile–0.1% phosphoric acid (1 : 99) into the residue to dissolve. Finally, filter it through a microporous membrane (0.45 μm) and obtain the filtrate.

### 3.6 Determination method

Inject 10 μL of the reference solution and test solution into HPLC, record the chromatogram.

### 3.7 Methodology

#### 3.7.1 Precision of the experiment

Take the reference solution and the test solution, and inject the sample for 6 times according to the chromatographic conditions (3.4). Then, calculate the peak area of triglochinic acid in both reference and test solution. The RSD is less than 2%, indicating that the instrument is precise.

#### 3.7.2 Repeatability test

Take 6 samples of the same batch of *Pinelliae pedatisectae rhizoma*, and accurately weigh 1 g. Follow the method in 3.5 to prepare the test solution and the chromatographic conditions in 3.4 for tests. The RSD of peak area of triglochinic acid was 0.57%, indicating that this method is repetitive.

#### 3.7.3 Stability test

Take the same test solution, and store it at room temperature. Follow the chromatographic method in 3.4 to test at 0 h, 4 h, 8 h, 12 h, 18 h and 24 h. The results showed that the RSD of peak area of triglochinic acid from test solution was 0.92%, indicating that the sample solution was relatively stable within 24 hours.

#### 3.7.4 Detection limit

Take the reference solution, and dilute it to 0.10 μg mL^−1^ with water. Accurately inject 10 μL into the HPLC, and calculate the signal-to-noise ratio (S/N = 3 as the detection limit). The detection limit was 545.5 μg kg^−1^ (Table 4).

**Table 4:** Detection limit^a^

Component	Concentration of the reference solution (μg mL^−1^)	Signal-to-noise ratio S/N	Dilution ration of the test solution	Sample amount (g)	Detection limit (μg kg^−1^)
Triglochinic acid	0.10	2.2	4	1.0000	545.5

a NB: detection limit (μg kg^−1^) = concentration of reference solution × injection volume of reference solution × 3 × dilution ratio of test solution/signal-to-noise ratio/sample amount/sample injection volume.

### 3.8 Sample tests

28 batches of *Pinelliae rhizoma*, 16 batches of *Pinelliae pedatisectae rhizoma* and 39 batches of commercial medicinal materials were prepared according to the method in 3.5, and determined following the chromatographic conditions in 3.4. If there is a peak consistent with the retention time of triglochinic acid, and the absorption spectrum of the corresponding chromatographic peak is the same within the wavelength range of 190–400 nm by the diode-array detector, the sample will be identified as *Pinelliae pedatisectae rhizoma*. The results were shown in Table 5.

**Table 5:** Triglochinic acid test results in each sample^a^

ID	Triglochinic acid	ID	Triglochinic acid	ID	Triglochinic acid	ID	Triglochinic acid
BX1	−	BX22	−	HZ15	+	SSBX20	+
BX2	−	BX23	−	HZ16	+	SSBX21	−
BX3	−	BX24	−	SSBX1	+	SSBX22	+
BX4	−	BX25	−	SSBX2	+	SSBX23	−
BX5	−	BX26	−	SSBX3	+	SSBX24	+
BX6	−	BX27	−	SSBX4	−	SSBX25	−
BX7	−	BX28	−	SSBX5	−	SSBX26	−
BX8	−	HZ1	+	SSBX6	+	SSBX27	+
BX9	−	HZ2	+	SSBX7	−	SSBX28	−
BX10	−	HZ3	+	SSBX8	−	SSBX29	+
BX11	−	HZ4	+	SSBX9	+	SSBX30	+
BX12	−	HZ5	+	SSBX10	+	SSBX31	+
BX13	−	HZ6	+	SSBX11	+	SSBX32	+
BX14	−	HZ7	+	SSBX12	+	SSBX33	+
BX15	−	HZ8	+	SSBX13	+	SSBX34	+
BX16	−	HZ9	+	SSBX14	+	SSBX35	−
BX17	−	HZ10	+	SSBX15	+	SSBX36	−
BX18	−	HZ11	+	SSBX16	+	SSBX37	−
BX19	−	HZ12	+	SSBX17	+	SSBX38	+
BX20	−	HZ13	+	SSBX18	+	SSBX39	+
BX21	−	HZ14	+	SSBX19	+		

a NB: “+” for triglochinic acid detected, “−” for not.

The results showed that triglochinic acid was not detected in the 28 batches of *Pinelliae rhizoma*, while detected in all the 16 batches of *Pinelliae pedatisectae rhizoma*. However, triglochinic acid were detected in 27 of the 39 batches of the commercial samples. Therefore, the rate of fake products of *Pinelliae rhizoma* was 69.2%.

### 3.9 HPLC-MS verification of triglochinic acid in the samples

#### 3.9.1 Liquid chromatographic conditions

The Agilent 1290-6460 LC/MS was used with Agilent ZORBAX Eclipse Plus C_18_ column (2.1 × 50 mm, 1.8 μm) and the mobile phase of methanol–0.02% ammonia solution (5 : 95) at 0.15 mL min^−1^ flow rate, 45 °C column temperature. The injection volume was 2 μL.

#### 3.9.2 Mass spectrometry conditions

Multi-reaction monitoring (MRM) was performed with a mass spectrometer detector, in electrospray negative ion mode (ESI^−^) at 3500 capillary voltage. Flow rate of dryer was 9 L per minute, temperature of dryer was 280 °C, 40 psi Nebulizer, and 0 V Fragmentor (secondary). The parameters of triglochinic acid were shown in Table 6.

**Table 6:** Multi-reaction detection of ion ratios for triglochinic acid

Parent ion	Daughter ion	Collision energy
187	143	5
187	99	9

#### 3.9.3 Preparation of the solution

In the preparation of the test solution, the acidifying reagent phosphoric acid was replaced by formic acid, while the remaining was as same as in 3.5.

#### 3.9.4 Results verification

The multi-reaction detection of ion ratios for triglochinic acid, *Pinelliae rhizoma*, and *Pinelliae pedatisectae rhizoma* samples were shown in Table 7. The molecular ion peaks of *Pinelliae pedatisectae rhizoma* and triglochinic acid were the same (187), which is consistent with the mass of triglochinic acid ion (C_7_H_7_O_6_^−^). The molecular ion peaks were further confirmed by MS/MS, showing that the secondary ions were the same: 143 and 99.

**Table 7:** Ion ratio of triglochinic acid in reaction monitoring

Sample name	Set ratio	Actual ratio
Triglochinic acid	96.37	96.37
*Pinelliae pedatisectae rhizoma*	96.37	93.38
*Pinelliae rhizoma*	96.37	—

According to the results of HPLC-MS, the peak retention time detected in *Pinelliae pedatisectae rhizoma* was consistent with that of triglochinic acid. Furthermore, the mass-to-charge ratio of the selected two pairs of daughter ions were consistent. The relative abundance of qualitative ions of *Pinelliae pedatisectae rhizoma* and triglochinic acid were within the range of tolerance (±20%),^14,15^ while their retention times were also consistent. Since the triglochinic acid peak was not detected in *Pinelliae rhizoma*, it can be verified that *Pinelliae rhizoma* does not contain triglochinic acid, while *Pinelliae pedatisectae rhizoma* does.

## 4. Discussion

*Pinelliae pedatisectae rhizoma* and *Pinelliae rhizoma* belong to the same genus, and share similar characteristics. The processed or young tubers of *Pinellia pedatisecta* are sold as *Pinelliae rhizoma* in herbal medicine market. While the identification has always been difficult to achieve. In addition, *Pinelliae pedatisectae rhizoma* in large size consists of the main tuber and a few small attached ones, resembling a tiger's claw.^16^ Due to the factors like provenance, soil, pests and diseases, and cultivation, some trait variations occur to *Pinelliae rhizoma* as well: one or several small tubers appear around the major tuber, which is similar to the characters of *Pinelliae pedatisectae rhizoma* and is easily misidentified as *Pinelliae pedatisectae rhizoma*. *Pinelliae rhizoma*, with its various forms of prepared drug in pieces, is popular in clinical use. On the market, the products of *Pinelliae pedatisectae rhizoma* are sold as the counterfeit of *Pinelliae rhizoma* because after processing, their color and surface characteristics further change. Especially after slicing, *Pinelliae rhizoma* and *Pinelliae pedatisectae rhizoma* are almost indistinguishable from their appearances.

The chemical constituents of *Pinelliae rhizoma* and *Pinelliae pedatisectae rhizoma* are similar. *Pinelliae pedatisectae rhizoma* contains a variety of alkaloids, dipeptides, amino acids, organic acids, nucleosides, and polysaccharides,^17–20^ the same does *Pinelliae rhizoma* except for dipeptides.^21–27^ At present, morphological identification, thin layer chromatography identification and fingerprint identification are the main identification methods for *Pinelliae rhizoma*. However, morphological identification faces great difficulty especially after processing or slicing. Chen *et al.*^28^ identified one more spot in *Pinelliae rhizoma* which was not detected in *Pinelliae pedatisectae rhizoma* by means of thin-layer chromatography, but this method could not determine whether *Pinelliae rhizoma* was incorporated or not. Lu^9^ established the fingerprints of *Pinelliae rhizoma* and *Pinelliae pedatisectae rhizoma*, in which *Pinelliae rhizoma* had three more chromatographic peaks than *Pinelliae pedatisectae rhizoma*, but it was also unable to identify the incorporated *Pinelliae rhizoma*.

Triglochinin, which can be hydrolyzed into triglochinic acid, was found in the flowers of *Triglochin maritima*,^29^ the young leaves of *Alocasia*^30,31^ and *Ranunculaceae* genus.^32^ The *Alocasia* genus is toxic, and the whole plant contains cyanogenic glycoside. However, whether cyanogenic glycoside is the main substance causing the toxicity of *Alocasia macrorrhizos* is not fully understood. According to the literatures, cyanogenic glycoside itself is not toxic, but it can be degraded by β-glucosidase and α-hydroxynitrile lyase, thereby releasing the toxic hydrogen cyanide (HCN) as well as glucose and aldehydes or ketones,^33^ resulting in toxic effects. The triglochinic acid was found in *Pinelliae pedatisectae rhizoma*, but whether it is toxic remains to be confirmed in future research. *Pinelliae rhizoma* and *Pinelliae pedatisectae rhizoma* are of the same genus *Araceae*, which is inherently toxic: mainly stimulating toxic effects, caused by the shared raphides and lectin proteins.^34^ Furthermore, their efficacy and clinical applications are different. Therefore, the use should be strictly differentiated, and the safety of *Pinelliae rhizoma* mixed with *Pinelliae pedatisectae rhizoma* should be concerned as well.

In addition, the history of artificial cultivation of *Pinellia ternata* is short. The seeds of *Pinellia ternata* are mostly wild, sometimes being mixed with that of *Pinellia pedatisecta*, resulting in a small amount of *Pinelliae pedatisectae rhizoma* identified in *Pinelliae rhizoma*. Now it is highly necessary to strengthen the research on the seeds standard of *Pinellia ternata*, and control its quality from the source.

In this study, for the first time we isolated the triglochinic acid from *Pinelliae pedatisectae rhizoma* and established the HPLC identification method and LC-MS verification method. This method is stable, accurate, and widely applicable. It can be used for the identification of *Pinelliae pedatisectae rhizoma* in *Pinelliae rhizoma* materials or prepared drugs. As a supplement to the quality control of *Pinelliae rhizoma* in the Chinese Pharmacopoeia, this method effectively combats the situation of adulteration and counterfeiting in the market, protecting the interests of farmers, planting enterprises, merchants, and companies. It also promotes the quality control of *Pinelliae rhizoma*, effectively ensuring the safety of clinical use.

## Conflicts of interest

There are no conflicts to declare.

## Supplementary Material
